# Target-site dynamics explain a large share of apparent microRNA differential expression

**DOI:** 10.1261/rna.080990.126

**Published:** 2026-07

**Authors:** Mert Cihan, Piyush More, Maximilian Sprang, Federico Marini, Miguel A. Andrade-Navarro

**Affiliations:** 1 Faculty of Biology, Institute of Organismic and Molecular Evolution, Johannes Gutenberg University Mainz, Mainz 55118, Germany; 2 Department of Dermatology, University Medical Center of the Johannes Gutenberg University Mainz, Mainz 55128, Germany; 3 Institute of Quantitative and Computational Biology, Johannes Gutenberg University Mainz, Mainz 55128, Germany; 4 Institute of Medical Biostatistics, Epidemiology and Informatics, University Medical Center Mainz, Mainz 55128, Germany; 5 Research Center for Immunotherapy (FZI) Mainz, Mainz 55113, Germany

**Keywords:** alternative polyadenylation, gene regulation, microRNA, noncoding RNA, target-directed microRNA degradation

## Abstract

MicroRNA (miRNA) abundance reflects a dynamic balance between biogenesis, target engagement, and decay, yet differential expression analyses typically ignore changes in target-site availability driven by alternative polyadenylation (APA). We introduce MIRNAPEX, an expression-stratification-based machine learning framework that quantifies miRNA regulatory effect sizes from RNA-seq data by integrating target-gene expression with 3′UTR isoform usage to infer effective binding-site dosage. Using pan-cancer training sets, we train models that learn relationships between transcriptomic features and miRNA log fold changes, with APA patterns providing context-dependent complementary information alongside gene expression. When applied to knockdowns of core APA regulators, MIRNAPEX captured widespread 3′UTR shortening and predicted miRNA-specific shifts whose direction was consistent with changes in the APA-associated 3′UTR landscapes of target genes. Analysis of target-directed miRNA degradation interactions further showed that loss of distal decay-trigger sites coincides with increased miRNA abundance, consistent with reduced target-directed miRNA degradation. Together, these findings suggest that apparent miRNA differential expression can be associated with dynamic target-site landscapes in addition to altered miRNA transcription, and that neglecting this dimension can lead to misestimation of regulatory effect sizes.

## INTRODUCTION

MicroRNAs (miRNAs) are short (∼22 nt) noncoding RNAs that posttranscriptionally regulate gene expression by binding to partially complementary sites in the 3′ untranslated regions (3′UTRs) of target genes (Bartel 2009). By doing so, miRNAs fine-tune developmental programs, buffer cellular stress responses, and, if dysregulated, contribute to diverse disease phenotypes, including metabolic disorders, cancer, and neurodegenerative diseases (Bartel 2018; O'Brien et al. 2018; Vaghf et al. 2022; Kapplingattu et al. 2025). Considering the essential role of miRNAs in maintaining normal physiology and their potential as predictive biomarkers (Kimura et al. 2023), accurate quantification of their expression level is crucial. In comparative transcriptomic analyses, differences in miRNA expression between conditions are commonly interpreted as indicators of altered posttranscriptional regulation and a reflection of broader regulatory state changes (Calin and Croce 2006; Lu and Clark 2012). However, the steady-state level of a mature miRNA reflects a moving balance of three broad processes that determine the cellular abundance of each miRNA species. First, biogenesis, which includes transcription of the primary transcript, Microprocessor cleavage, nuclear export, Dicer processing, and loading of Argonaute (AGO) proteins to form RNA-induced silencing complexes (RISCs), sets the potential pool of mature miRNAs (O'Brien et al. 2018; Kim et al. 2025). Second, target engagement redistributes miRNA–RISC complexes across the transcriptome and determines how strongly a given miRNA can repress its targets in a particular cellular state (Jonas and Izaurralde 2015; Bartel 2018; McGeary et al. 2019). Third, decay pathways remove mature miRNAs, with general turnover mechanisms such as tailing and trimming followed by exonucleolytic decay controlling their half-life (Eichhorn et al. 2014; Bofill-De Ros and Vang Ørom 2024).

In addition, target-directed miRNA degradation (TDMD) is a process in which binding to a highly complementary target transcript actively triggers destabilization and decay of the miRNA itself, thereby accelerating its decay. For instance, systematic AGO-CLASH analyses have revealed numerous endogenous transcripts that act as TDMD triggers, indicating that target-directed degradation of miRNAs is more prevalent than previously recognized (Ameres et al. 2010; Li et al. 2021b; Buhagiar and Kleaveland 2024). Because biogenesis, target engagement, and decay, including TDMD, act simultaneously and dynamically, observed changes in miRNA abundance may not directly report transcriptional output but can reflect shifts in target availability and turnover.

Accordingly, several studies have estimated miRNA activity from properties of their targets, showing that target gene abundance and site affinity predict miRNA levels, AGO binding, and competition effects (Cheng and Li 2008; Bosson et al. 2014; Diener et al. 2024; Cihan et al. 2025a,b). Intuitively, target expression sets the demand placed on a miRNA, such that abundant, site-rich targets increase demand, whereas depletion of those targets reduces it (Bosson et al. 2014; Denzler et al. 2014, 2016). However, most target-centric approaches treat each gene as if it had a single, fixed 3′UTR, overlooking the widespread phenomenon of alternative polyadenylation (APA) (Tian and Manley 2017). APA leads to the generation of transcript isoforms with distinct 3′UTR lengths and can therefore add or remove canonical miRNA binding sites as well as highly complementary TDMD trigger sites, dynamically altering the effective binding-site dosage available to each miRNA. Indeed, more than half of human genes utilize APA to generate alternative 3′UTR isoforms, meaning that dynamic 3′UTR length changes broadly modulate available miRNA binding sites and thus influence posttranscriptional regulation (Tian and Manley 2017; Fu et al. 2018; Marini et al. 2021; Cihan et al. 2025c). Since APA is a widespread mechanism that controls 3′UTR length and miRNA-site availability, perturbation experiments of core APA factors (such as CFIm25, CFIm68, and CPSF6) have shown that knocking them down remodels thousands of 3′UTRs across the transcriptome (Masamha et al. 2014; Ghosh et al. 2022). Such APA-driven alterations in miRNA targeting have been shown to impact gene expression programs, stem cell function and differentiation, and oncogenic transformation, among others, highlighting the crucial interplay between APA and miRNA regulation in fine-tuning cellular phenotypes (Sandberg et al. 2008; Boutet et al. 2012).

Despite this, most differential expression (DE) analyses of miRNAs ignore dynamic changes in effective target-site dosage caused by APA and their impact on TDMD, creating a gap in how observed miRNA shifts are interpreted. We therefore hypothesize that APA-driven 3′UTR remodeling, by altering binding-site availability, can contribute to associations between target-site dosage and observed changes in mature miRNA abundance. In this framework, effective target-site availability may correlate with the effect size of miRNA DE, typically quantified as log fold change (logFC), between states and samples. Consequently, apparent miRNA DE may partly reflect changes in target-site landscapes in addition to altered miRNA transcription.

Motivated by this gap, we introduce MIRNAPEX, an expression-stratification-based interpretable machine learning (ML) framework that predicts miRNA logFC from RNA-seq by integrating target-gene expression with 3′UTR isoform usage to estimate effective binding-site dosage. Beyond prediction, we quantify the relative impact of APA variation on each miRNA and apply MIRNAPEX to APA-factor perturbation data sets to evaluate whether global 3′UTR shortening is associated with predictable shifts in miRNA levels. We further examine curated TDMD trigger–miRNA pairs to see if loss of distal TDMD sites coincides with expected increased miRNA abundance (Buhagiar and Kleaveland 2024). Altogether, this approach shows how transcriptomic variation, including 3′UTR remodeling, shapes miRNA abundance, underscoring that miRNA DE and its estimated effect size should be interpreted in the context of dynamic target-site landscapes.

## RESULTS

### Transcriptomic prediction of miRNA expression changes

miRNA logFC values between sample groups were predicted using features derived from their putative target genes. Two types of features were considered: gene-level logFC values, reflecting differential mRNA expression, and ΔPDUI values, capturing changes in APA patterns. Together, these measures serve as proxies for the relative abundance of miRNA binding sites within their target genes. To avoid reliance on a single modeling assumption, we compared several commonly used regression algorithms that differ in their treatment of high-dimensional feature spaces. These include linear models with L1 or L2 regularization, which emphasize feature selection or coefficient shrinkage, as well as nonlinear models that capture complex relationships through ensemble or kernel-based approaches. To assess predictive performance, ML algorithms were built using feature sets of varying size, defined by ranked microT interaction scores (see Materials and Methods for details).

Linear models substantially outperformed nonlinear approaches in predicting miRNA logFC values across feature set sizes (Fig. 2A). EN achieved the highest mean *R*^2^, followed closely by LA and RI, while nonlinear models such as RF, HB, and MLP performed worse. As a baseline algorithm, OLS exhibited a marked decline in performance once the feature set exceeded ∼250 genes, highlighting the importance of regularization in high-dimensional settings. These findings are consistent with prior observations that linear models are well suited for modeling miRNA expression dynamics (Cihan et al. 2025b).

**FIGURE 1.**
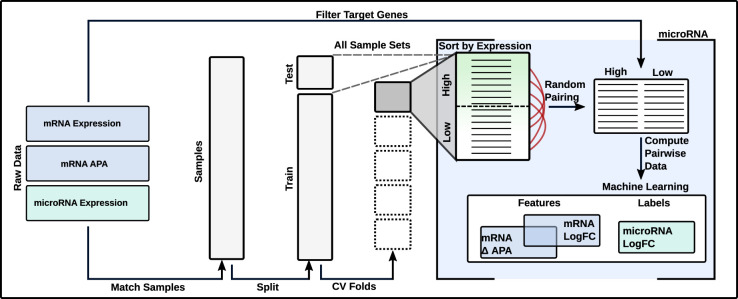
Workflow for training ML models to predict miRNA logFC. Matched TCGA samples containing mRNA expression, APA profiles, and miRNA expression are split into random training (80%) and test (20%) sets, with the training set further divided into cross-validation folds. Within each fold, samples are stratified by miRNA expression levels and randomly paired in both directions to prevent data leakage during training. For each miRNA, annotated target genes with valid APA measurements are selected, and differential features (mRNA logFC, ΔPDUI) are computed for each sample pair. The observed miRNA logFC serves as the prediction label, and the same feature computation is applied across folds, the test set, and the final training set after hyperparameter tuning. A separate ML model is trained for each miRNA to capture the relationship between transcriptomic changes and miRNA expression dynamics.

**FIGURE 2.**
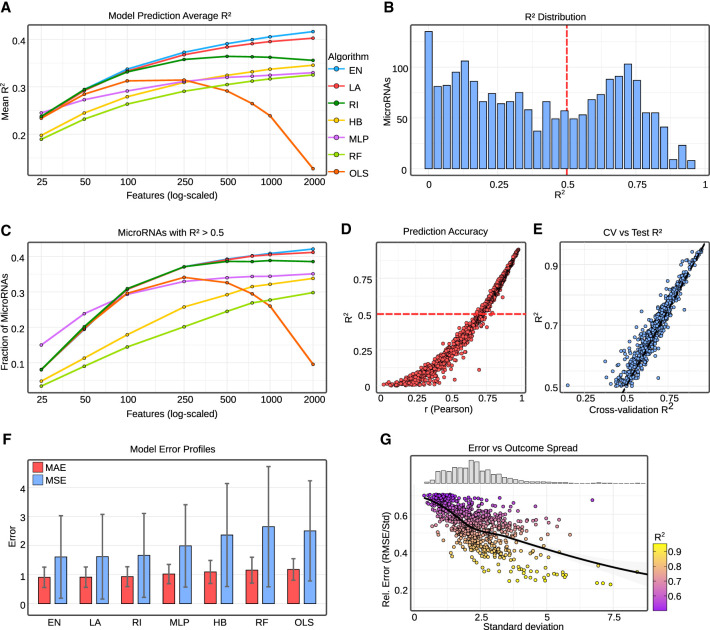
Prediction performance of ML models for miRNA logFC. (*A*) Line plot of mean *R*^2^ values across different ML algorithms as a function of the number of input features (tick marks represent logarithmically spaced values). Algorithms are abbreviated as follows: (EN) elastic net, (LA) lasso, (RI) ridge regression, (HB) HistGradientBoost, (MLP) multilayer perceptron, (RF) random forest, (OLS) ordinary least squares. (*B*) Distribution of *R*^2^ values from EN trained with 1000 features across all evaluated miRNAs. (*C*) Fraction of miRNAs achieving *R*^2^ > 0.5 as a function of the number of input features (tick marks represent logarithmically spaced values). (*D*) Dot plot of Pearson correlation between predicted and observed logFC values versus miRNA-specific *R*^2^. (*E*) Comparison of cross-validation *R*^2^ against test set *R*^2^ across miRNAs. (*F*) Mean absolute error (MAE) and mean squared error (MSE) across algorithms (for 1000 features). (*G*) Relationship between the standard deviation of predicted logFC values and relative error (RMSE/standard deviation). The distribution of standard deviation values is shown as a histogram.

A feature set size of 1000 was selected as the optimal balance between predictive accuracy and interpretability (Supplemental Table 1). At this scale, EN achieved a mean *R*^2^ of 0.41 across all miRNAs, and the distribution of prediction accuracies showed that 817 miRNAs (41%) surpassed the *R*^2^ > 0.5 threshold, with a mean *R*^2^ of 0.69 for this subset (Fig. 2B,C). To define highly predictable miRNAs (HP-miRNAs), we used a threshold of *R*^2^ > 0.5, corresponding to models that explain more than half of the observed variance. This cutoff aligns with the right-hand tail of the *R*^2^ distribution (Fig. 2B). These HP-miRNAs were prioritized for downstream analyses. Across all miRNAs, the average Pearson correlation between predicted and observed logFC values was 0.62, while the correlation increased to 0.83 for HP-miRNAs (Fig. 2D). Robustness of the models was further supported by the strong concordance between cross-validation and test set performance, with a Pearson correlation of 0.98 (Fig. 2E), indicating minimal over- or underfitting.

To further benchmark model accuracy, prediction errors were compared across algorithms at the 1000-feature setting (Fig. 2F). EN achieved the lowest mean absolute error (0.91) and mean squared error (1.60), further highlighting its robustness relative to the other methods. Moreover, analysis of relative error revealed that prediction error scaled proportionally to the variance of the observed logFC values and remained small relative to the standard deviation, particularly for HP-miRNAs (Fig. 2G).

Together, these analyses demonstrate that effect size estimate of the miRNA expression regulation can be predicted with high accuracy and robustness from transcriptomic features.

### Expression- and APA-driven signals jointly shape miRNA prediction accuracy

The prediction of miRNA activity from transcriptomic data has traditionally been based on mRNA expression levels measured by RNA-seq or microarray platforms (Nielsen and Pedersen 2021; Olgun et al. 2022). To investigate the added predictive value of 3′UTR patterns, we evaluated the role of APA. Specifically, we trained ML models for each miRNA using three different feature sets: expression-only, APA-only, and combined expression plus APA features.

**FIGURE 3.**
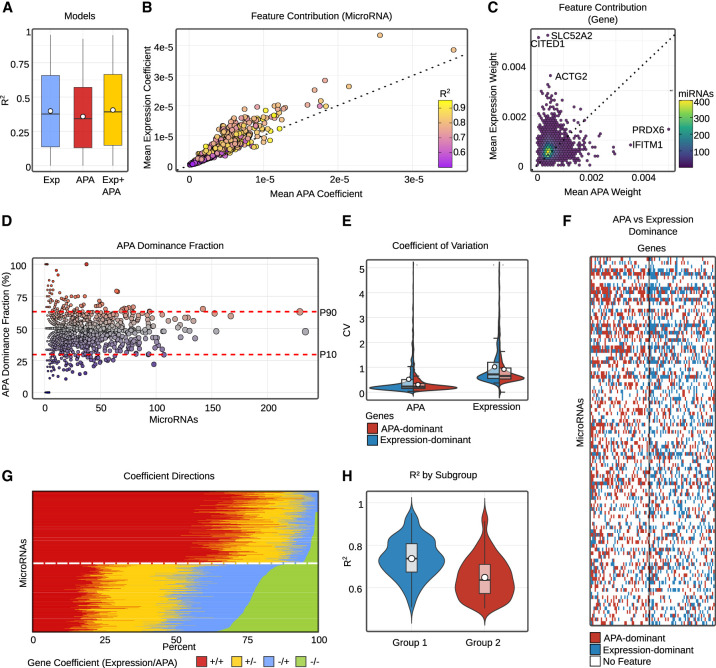
Contribution of APA and expression features to miRNA logFC prediction. (*A*) Comparison of predictive performance for models trained with APA-only, expression-only, or combined features. Boxplots show the distribution of *R*^2^ scores across miRNAs. (*B*) Scatter plot of average normalized absolute coefficients for APA versus expression features across miRNAs with *R*^2^ > 0.3. Each point represents one miRNA, colored by predictive performance (*R*^2^). (*C*) Hexbin plot of gene-level contributions, showing mean percentage weight of APA versus expression features across highly predictable miRNAs (*R*^2^ ≥ 0.5). Color scale denotes the number of miRNAs in which a given gene contributes. (*D*) Scatter plot of APA dominance fraction versus gene prevalence across all genes contributing to miRNAs with *R*^2^ ≥ 0.5. Each point represents one gene, with dashed lines marking the 10th and 90th percentile cutoffs used to define expression-dominant versus APA-dominant gene sets. (*E*) Coefficient of variation for APA versus expression features within the extreme APA-dominated and expression-dominated gene deciles. These measurements represent the raw variability from which APA and expression features are derived for model training. Stars denote outliers above the plotted range. (*F*) Heat map of categorical dominance (APA vs. expression) across all miRNAs with *R*^2^ ≥ 0.5 and the 100 most prevalent genes. Rows are ordered by decreasing miRNA *R*^2^ and columns by decreasing gene prevalence. White indicates that the gene was not a selected feature for that miRNA, blue indicates higher absolute expression coefficient, and red indicates higher absolute APA coefficient. (*G*) Stacked barplots of sign concordance between APA and expression coefficients across all genes for miRNAs with *R*^2^ ≥ 0.5. Colors denote the four possible sign combinations: both positive (++), APA positive with expression negative (+−), APA negative with expression positive (−+), and both negative (−−). miRNAs are stratified into two groups based on their composition, using a threshold of 10% (−−). (*H*) Comparison of *R*^2^ values between the two stratified groups.

Expression-only models achieved a mean *R*^2^ of 0.39 across all miRNAs, while APA-only models performed slightly lower with a mean *R*^2^ of 0.36. Importantly, the combined models improved performance to a mean *R*^2^ of 0.41, demonstrating that APA contributes complementary predictive information beyond gene expression alone (Fig. 3A). Among the HP-miRNAs, there were 802 miRNAs for the expression-only and 617 for the APA-only models scoring with *R*^2^ > 0.5. Although the gain in overall prediction performance when using expression and APA features together is modest compared with using either feature set alone, this analysis demonstrates two important points. First, APA-only models perform comparably to expression-only models, indicating that a measure independent of gene expression quantification, namely 3′UTR length patterns, can predict differential miRNA behavior. Consistent with this interpretation, stratified analysis showed that the benefit of adding APA features was most pronounced for miRNAs with low predictive performance in the expression-only model, indicating that APA contributes predictive signal primarily in cases where expression alone is insufficient (Supplemental Fig. 7).

Second, combining expression and APA features provides a unified framework to assess their relative and context-dependent contributions to miRNA regulation. To assess model performance on high-confidence miRNAs, we evaluated MIRNAPEX predictions for MirGeneDB-supported miRNA genes (Clarke et al. 2025). Across all MirGeneDB miRNA entries, the mean predictive accuracy was *R*^2^ of 0.61. At the gene level, allowing either mature arm to contribute, 364 of 506 MirGeneDB miRNA genes showed strong predictability (*R*^2^ > 0.5; Supplemental Fig. 8). Together, these results indicate that MIRNAPEX performance is strongest for high-confidence miRNA annotations and is not driven by low-confidence miRBase entries.

To further investigate the predictive signal, we examined feature contributions from both the miRNA and target gene perspectives.

From the miRNA perspective, analysis of average coefficients confirmed that both expression- and APA-derived features contributed substantially to prediction accuracy, with no miRNA relying exclusively on a single modality (Fig. 3B). Expression features were moderately more influential overall, with 77% of miRNAs showing higher weights for expression than for APA. This bias, however, was modest rather than extreme, and no outliers exhibited complete dependence on one feature type, consistent with previous observations.

From the gene perspective, we assessed whether target genes contributed systematically through expression or APA features. Among 8260 target genes across all HP-miRNAs, 70% exhibited a bias toward expression-derived contributions. Specific examples included *CITED1*, *SLC52A2*, and *ACTG2*, which were primarily expression-driven, whereas *IFITM1* and *PRDX6* were dominated by APA. Nonetheless, most genes featured in many miRNA models (>200) showed no strong preference, again highlighting the balanced contributions of both modalities (Fig. 3C).

To further dissect modality-specific contributions, we stratified genes into APA- and expression-dominant groups based on the 10th and 90th percentile cutoffs of their dominance fraction. This classification yielded 891 APA-dominant and 768 expression-dominant genes across all HP-miRNAs. Notably, genes with high prevalence across multiple miRNAs typically exhibited only moderate dominance (Fig. 3D).

When comparing variability across modalities, expression-dominant genes showed higher variance in both expression and APA relative to APA-dominant genes. For expression values, median coefficients of variation were 0.716 versus 0.650, and for APA, 0.225 versus 0.221. A Wilcoxon rank-sum test confirmed significantly greater variability in expression (*P* < 0.001) and APA (*P* < 0.01) for expression-dominant genes. Importantly, APA-dominant genes did not exhibit elevated APA variability across miRNAs, indicating that their predictive contribution reflects systematic APA regulation rather than noise (Fig. 3E). Similarly, the top 100 recurrently used genes were rarely exclusive to APA or expression, but instead reflected mixed contributions (Fig. 3F).

### Bimodal sign patterns reveal distinct expression–APA relationships

Beyond their relative magnitudes, the coefficient signs for expression- and APA-derived features reveal how these two modalities tend to covary within our models. In general, positive coefficients for both expression and APA of target genes indicate that higher expression together with more distal 3′UTR usage is statistically associated with higher predicted miRNA levels, whereas negative coefficients for both modalities indicate the opposite: lower expression combined with more proximal site usage is statistically associated with lower predicted miRNA levels. These associations reflect predictive relationships and do not imply a specific direction of causality.

To assess whether individual miRNAs exhibit systematic patterns in how expression- and APA-derived contributions relate across their target genes, we summarized the distribution of coefficient sign combinations separately for each miRNA. miRNAs were then stratified according to whether concordant (++ and −−) or discordant (+− and −+) sign patterns predominated among their targets. This grouping was introduced to distinguish miRNAs for which expression and 3′UTR architecture tend to act in the same direction from those in which the two modalities contribute in opposing directions.

Applying this stratification revealed two dominant groups of miRNAs. About 423 HP-miRNAs (52%) were dominated by the concordant sign patterns (++ and −−), in which expression and APA coefficients share the same sign. The remaining 390 HP-miRNAs (48%) were dominated by the discordant sign patterns (+− and −+), in which coefficients have opposite signs (Fig. 3G).

This bimodality highlights two prevalent modes by which expression and APA features relate to miRNA levels. Interestingly, these two groups also differed in predictive performance, with mean *R*^2^ values of 0.74 and 0.65, respectively (Wilcoxon rank-sum test, *P* < 0.01; Fig. 3H). While the coefficients come from regularized models and cannot be interpreted as direct effect sizes, the systematic separation is consistent with opposing mechanisms such as compensatory biogenesis versus target-directed degradation. However, because miRNA–target interactions are intrinsically bidirectional, increased target expression and 3′UTR lengthening may coincide with either higher or lower mature miRNA abundance, and the present framework cannot distinguish whether observed associations reflect dominant target-mediated sequestration, miRNA-driven repression, or a combination of both. Importantly, this does not diminish the biological relevance of the observed patterns, as the reproducible contribution of APA and expression features demonstrates that dynamic changes in target-site availability are associated with steady-state miRNA levels.

### Associations between APA regulation and miRNA logFC

To examine how APA modulates miRNA expression dynamics, we analyzed four perturbation experiments in which key APA-regulatory proteins were knocked down and compared with matched controls. These data sets included knockdowns of CFIm25 (two independent experiments), CFIm68, and CPSF6, factors that shape 3′UTR processing and thereby influence miRNA binding-site availability (Tian and Manley 2017). For each data set, we applied the MIRNAPEX pipeline to predict miRNA log fold changes based solely on the observed gene-expression changes and APA shifts of their target genes.

**FIGURE 4.**
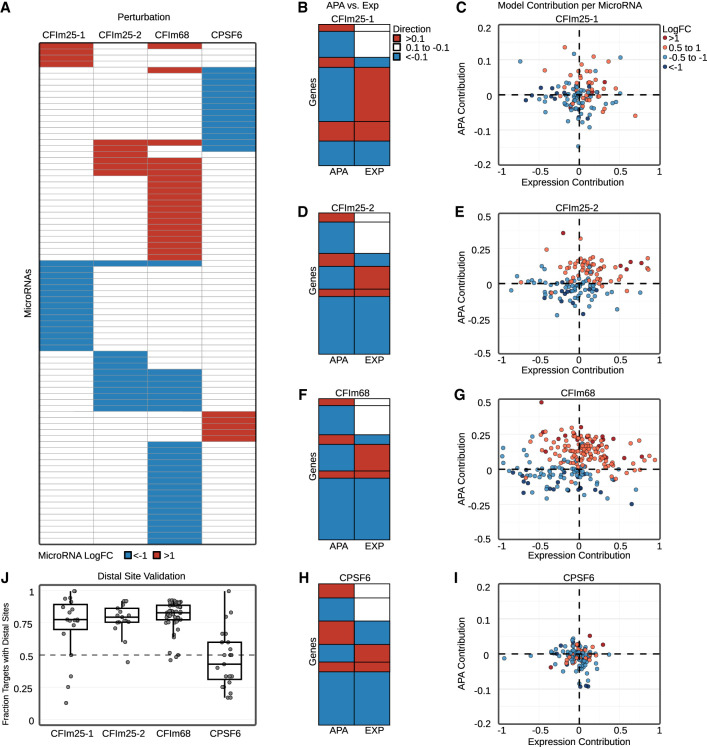
miRNA behavior in APA perturbation experiments. (*A*) Heat map of MIRNAPEX-computed miRNA logFC across the four knockdown (KD) experiments of APA-regulatory factors (CFIm25-KD-1, CFIm25-KD-2, CFIm68-KD, and CPSF6-KD). Only miRNAs with |logFC| >1 are displayed. (*B*,*D*,*F*,*H*) Heat maps display, for each gene with an APA change of |ΔPDUI| >0.1, the direction of 3′UTR change (APA column) together with the corresponding gene logFC (EXP column). Lengthening of 3′UTRs or positive gene logFC values are indicated in red, shortening or negative gene logFC values in blue, and no expression change in white. This representation highlights the directionality of APA changes relative to gene expression, showing whether 3′UTR lengthening or shortening coincides with increases or decreases in gene logFC across APA perturbation experiments. (*C*,*E*,*G*,*I*) Scatter plots show, for each miRNA across the perturbation experiments, the combined (unscaled) contribution of gene expression changes versus APA changes to the predicted miRNA logFC as computed by MIRNAPEX. Points are shaded according to the predicted miRNA logFC values across the defined thresholds. (*J*) Boxplots showing the proportion of APA-changed target genes that harbor at least one distal binding site for the same miRNA (|logFC| >1) in each perturbation experiment.

Across all four perturbation experiments (CFIm25-KD-1, CFIm25-KD-2, CFIm68-KD, and CPSF6-KD), we observed predicted miRNA logFC in both directions, with many exceeding an absolute value of 1 (Fig. 4A). In CFIm25-KD-1 (four miRNAs upregulated and 15 downregulated), CFIm25-KD-2 (six up and 11 down), CFIm68-KD (20 up and 25 down), and CPSF6-KD (six up and 14 down), the MIRNAPEX predictions indicated a range of miRNA logFC rather than a uniform shift. Notably, hsa-miR-182-5p showed consistent downregulation in three of the four experiments.

Since the direction of individual miRNA changes correlated with the expression and APA shifts of their target genes, we investigated how many genes with APA changes also display corresponding differences in gene expression and how the direction of 3′UTR change differences relates to the gene logFC to reveal the directionality of these effects.

Across the four perturbation experiments, we observed widespread changes in 3′UTR usage, with a clear predominance of shortening events and varying degrees of buffering, expression changes in the opposite direction to the APA effect, likely reflecting compensatory mechanisms such as altered miRNA activity as a consequence of binding site modulation.

In CFIm25-KD-1, 6721 genes displayed altered 3′UTR usage (defined as ΔPDUIs ≥0.05 for genes with |logFC| ≥0.1), with 5325 (79%) showing shortening; about 1698 (25%) of these APA-changed genes exhibited opposite (buffering) expression shifts (Fig. 4B). In CFIm25-KD-2, 385 genes showed altered 3′UTR usage, with 286 (74%) showing shortening and 175 (46%) displaying opposite expression changes (Fig. 4D). In CFIm68-KD, 6902 genes had altered 3′UTR usage, with 5742 (83%) showing shortening and roughly 1767 (26%) exhibiting opposite expression changes (Fig. 4F). In CPSF6-KD, 361 genes displayed altered 3′UTR usage, with 240 (67%) showing shortening and about 105 (29%) showing opposite expression changes (Fig. 4H).

We tested whether APA remodeling translates into predictable shifts in miRNA levels by splitting each miRNA's predicted change into two additive components: one reflecting expression of target genes and the other reflecting APA patterns, keeping unscaled values to highlight the direction and relative magnitude.

We found that miRNAs with larger predicted changes cluster in quadrants where the APA component change and the observed miRNA change point in the same direction. In CFIm25-KD-1, CFIm25-KD-2, and CFIm68-KD, this concordance is significant (one-sided Fisher's exact test, *P* < 0.01 for miRNAs with |logFC|>0.5), indicating that stronger APA-related signals are associated with larger predicted miRNA shifts (Fig. 4C,E,G). In CPSF6-KD, no enrichment is observed (*P* = 0.53), consistent with this perturbation showing the lowest fraction of 3′UTR-shortened genes among the data sets considered (Fig. 4I). This pattern further emphasizes that extensive gene shortening in APA perturbations coincides with the largest shifts in miRNA levels and that, where shortening is limited, the APA component contributes less strongly to miRNA log fold changes.

For each miRNA with a predicted change, we assessed whether the 3′UTR shortening or lengthening of its target genes in the same experiment is associated with altered availability of binding sites for that specific miRNA. Using the microT predictions, APA-changed genes were screened for the presence of at least one binding site for the same miRNA in the region between the proximal and distal polyadenylation sites. This analysis showed that in CFIm25-KD-1, CFIm25-KD-2, and CFIm68-KD, a substantial fraction of shortened targets indeed contained a distal binding site for the same miRNA, with median values of 77.8%, 79.7%, and 83.1% of APA-changed genes, respectively. In CPSF6-KD, the median proportion was much lower (42.9%), consistent with the weaker shortening seen in this data set (Fig. 4J). These results support the interpretation that predicted miRNA shifts are associated with changes in binding-site dosage and APA remodeling. To further validate PDUI as a proxy for miRNA binding-site availability, we extended this analysis to all expressed miRNAs in each perturbation data set, independent of their predicted logFC. Across data sets, APA-regulated target genes frequently harbored distal binding sites for the corresponding miRNA, with median fractions of 72.7% in CFIm25 KD–HCT116, 70.7% in CFIm25 KD–HEK293, 70.7% in CFIm68 KD–HEK293, and 56.5% in CPSF6 KD–HEP3B. These genome-wide results support PDUI as a meaningful measure of miRNA binding-site dosage in the present analyses.

In summary, our findings show a strong statistical concordance between APA-driven target shortening and miRNA logFC. This suggests that at least part of the apparent miRNA DE we observe may reflect changes in binding-site availability rather than direct changes in miRNA transcription.

### APA-driven loss of TDMD trigger sites coincides with miRNA abundance shifts

As APA-perturbation experiments globally shorten 3′UTRs, we asked whether this remodeling also affects established TDMD interactions. We therefore investigated eight curated trigger–miRNA pairs, as well as one negative control, in which the trigger transcript harbors highly complementary sites known to direct miRNA decay and for which we observed APA changes of the trigger gene. These included *CYRANO* with hsa-miR-7-5p, *SDC2* with hsa-miR-15a-5p, *SERTAD3* with hsa-miR-92a-3p, *SSR1* with hsa-miR-218-5p, *TRIM9* with hsa-miR-218-5p, *TDP1* with hsa-miR-320a-3p, *NREP* with hsa-miR-29b-3p, and *BCL2L11* with hsa-miR-221-3p, alongside *BCL2L11* with hsa-miR-221-5p as a negative control (Kleaveland et al. 2018; Li et al. 2021b, 2025).

**FIGURE 5.**
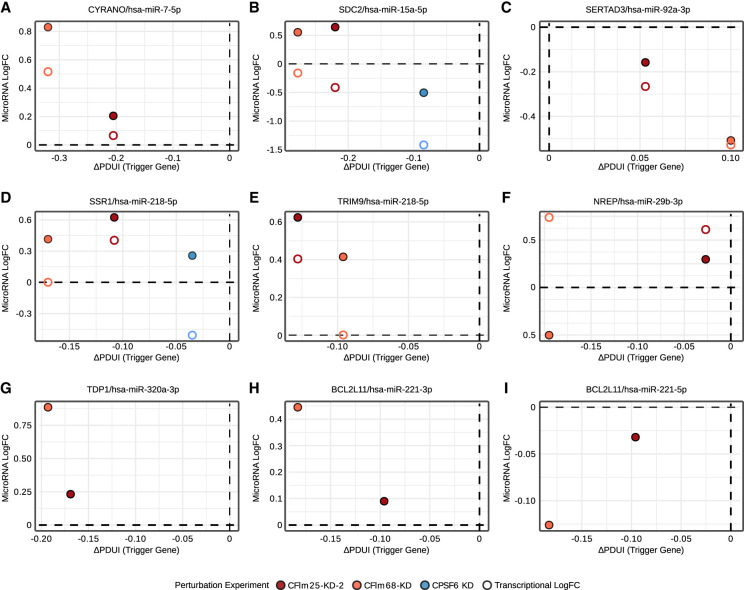
APA-driven 3′UTR shortening of TDMD trigger genes and miRNA abundance changes. Scatter plots relating APA changes of TDMD trigger genes to the log fold change of their paired mature miRNAs across APA-perturbation data sets. Each panel corresponds to one trigger–miRNA pair: (*A*) *CYRANO* with hsa-miR-7-5p, (*B*) *SDC2* with hsa-miR-15a-5p, (*C*) *SERTAD3* with hsa-miR-92a-3p, (*D*) *SSR1* with hsa-miR-218-5p, (*E*) *TRIM9* with hsa-miR-218-5p, (*F*) *NREP* with hsa-miR-29b-3p, (*G*) *TDP1* with hsa-miR-320a-3p, (*H*) *BCL2L11* with hsa-miR-221-3p, and (*I*) *BCL2L11* with hsa-miR-221-5p (negative control). Filled symbols indicate mature miRNA logFC values predicted by MIRNAPEX, while open symbols represent transcriptional proxy logFC estimates derived from host-gene intronic RNA-seq signal. Points are colored by perturbation data set. The *x*-axis shows ΔPDUI values (KD − control), with negative values indicating 3′UTR shortening, and the *y*-axis shows log fold changes.

For each APA-perturbation data set, we extracted the trigger genes, quantified their ΔPDUI values to assess 3′UTR remodeling, and compared these changes with MIRNAPEX-predicted log fold changes of the corresponding mature miRNAs (Fig. 5A–I). This analysis examines whether loss of distal 3′UTR regions harboring TDMD trigger sites is associated with increased abundance of the targeted miRNAs.

Across the majority of TDMD pairs, negative ΔPDUI values of the trigger gene, indicative of 3′UTR shortening, coincided with increased mature miRNA abundance. This trend was particularly evident for pairs involving *CYRANO* with hsa-miR-7-5p (Fig. 5A), *SDC2* with hsa-miR-15a-5p (Fig. 5B), *SSR1* with hsa-miR-218-5p (Fig. 5D), *TRIM9* with hsa-miR-218-5p (Fig. 5E), *TDP1* with hsa-miR-320a-3p (Fig. 5G), and *BCL2L11* with hsa-miR-221-3p (Fig. 5H). In these cases, the strongest miRNA upregulation was observed in data sets exhibiting pronounced trigger 3′UTR shortening, consistent with reduced TDMD-mediated degradation following loss of distal trigger regions (Buhagiar and Kleaveland 2024). In contrast, *SERTAD3* with hsa-miR-92a-3p (Fig. 5C) did not exhibit consistent 3′UTR shortening across perturbations and accordingly showed decreased miRNA abundance in conditions associated with 3′UTR lengthening, supporting the directional relationship between trigger 3′UTR architecture and miRNA stability. *NREP* with hsa-miR-29b-3p (Fig. 5F) deviated from the general trend, displaying divergent miRNA responses across perturbations despite trigger shortening, suggesting that additional regulatory inputs or context-dependent effects may modulate TDMD efficiency for this pair.

Importantly, the negative control pair *BCL2L11* with hsa-miR-221-5p (Fig. 5I) did not show systematic miRNA upregulation despite APA changes of the trigger gene, indicating that TDMD sensitivity is arm-specific and reinforcing that the observed effects are not a general consequence of trigger gene expression changes.

To distinguish posttranscriptional effects from altered miRNA production, we additionally compared mature miRNA logFC values with transcriptional proxy measurements derived from host-gene intronic RNA-seq signal (hollow circles in Fig. 5A–F). For all TDMD pairs showing increased mature miRNA abundance under trigger shortening, the transcriptional proxy logFC was lower or unchanged, indicating that the observed miRNA upregulation cannot be explained by increased transcription. In contrast, *NREP* with hsa-miR-29b-3p showed no consistent separation between transcriptional proxy and mature miRNA changes, in line with its context-dependent behavior.

Taken together, these results suggest that MIRNAPEX predictions are consistent with TDMD-linked miRNA behavior in the majority of curated cases and that APA-driven loss of distal trigger regions is frequently associated with increased mature miRNA abundance independent of transcriptional changes. This supports the view that a substantial fraction of miRNA expression changes observed under APA perturbation reflects altered TDMD site availability rather than solely changes in miRNA transcription.

## DISCUSSION

Steady-state miRNA levels arise from a dynamic balance of biogenesis, target engagement, and decay, including TDMD, yet DE of miRNAs is often interpreted as evidence of altered posttranscriptional regulation (McGeary et al. 2019; Bofill-De Ros and Vang Ørom 2024; Buhagiar and Kleaveland 2024). Within this balance, APA reshapes 3′UTR isoform usage and therefore affects effective dosage of canonical binding sites and highly complementary decay triggers (Mayr 2017; Tian and Manley 2017). To test whether such transcriptome remodeling predicts miRNA logFCs between conditions, we developed MIRNAPEX, which reads out target-centric features derived jointly from gene expression changes and APA.

Across miRNAs, adding APA-derived features to expression-based models resulted in only a modest overall increase in predictive performance, indicating that expression and APA capture partly overlapping information. This is expected, as both feature sets are derived from the same transcriptome. However, the contribution of APA was not uniform across miRNAs. In a stratified analysis, the combined model outperformed the expression-only model most frequently for miRNAs with low expression-only predictive performance, whereas the benefit was smaller for miRNAs already well predicted from expression alone. Thus, APA does not act as a broadly orthogonal predictor, but instead provides complementary information in a subset of cases. Mechanistically, this is consistent with the idea that site dosage and binding strength together determine AGO occupancy and repression efficacy (Broderick et al. 2011; Elkayam et al. 2012; Smibert et al. 2013).

From a gene-centric perspective, many targets contributed to prediction mainly through expression features, reflecting changes in total abundance and baseline miRNA binding site load, while others were dominated by APA, consistent with isoform switches that add or remove distal sites or decay triggers without large changes in total transcript levels (Tian and Manley 2017).

Both target-gene expression and APA influence predicted miRNA logFC: Expression dominates overall, but APA leads for many targets. Interestingly, miRNAs fall into two behavior classes: In one, expression and APA effects align, more transcripts and longer 3′UTRs go with higher miRNA levels, and in the other, they oppose, so increased site availability is associated with lower miRNA. This bimodal pattern suggests distinct regulatory modes for different miRNAs, not just variation in target abundance and that such modes may be consistent with competition and sequestration effects and observed differences in miRNA–mRNA network behavior in recent studies (Mao et al. 2020).

Applying MIRNAPEX to experimental data where core APA regulators were knocked down revealed broad 3′UTR shortening, consistent with the known global impact of APA perturbations, with variable buffering at the expression level and predicted miRNA changes in both directions (Masamha et al. 2014; Zhu et al. 2018; Liu et al. 2023). Decomposing predictions showed that the largest miRNA shifts clustered where the APA contribution and miRNA direction agreed, especially in perturbations that induce extensive shortening, whereas when shortening was limited, the contribution was weaker. Screening shortened targets confirmed that a substantial majority harbored distal sites for the same miRNA in these data sets, validating that MIRNAPEX effects stem from site dosage rather than spurious correlations.

For TDMD trigger–miRNA pairs such as *BCL2L11*–miR-221-3p, *NREP*–miR-29b-3p, *SSR1*–miR-218-5p, *TDP1*–miR-320a-3p, and *TRIM9*–miR-218-5p, 3′UTR shortening generally coincided with higher predicted miRNA abundance, consistent with relief from decay (Li et al. 2021b; Buhagiar and Kleaveland 2024). TDMD triggers located in the 3′UTR have been shown to degrade miRNAs more effectively than identical triggers placed in coding sequences (Li et al. 2025), which supports our focus on 3′UTR site loss in interpreting miRNA changes. A recent study found an endogenous TDMD trigger with minimal noncanonical 3′-end base-pairing that is nevertheless sufficient to induce degradation of the miR-279 family (Hiers et al. 2025). This suggests that many APA-associated miRNA expression changes could reflect widespread but previously uncharacterized TDMD triggers captured by MIRNAPEX. These findings further indicate that MIRNAPEX is sensitive to mechanistically defined decay events embedded within broader APA remodeling.

By integrating gene expression and APA features derived from RNA-seq data, the framework enables prediction of miRNA log fold changes even in the absence of matched small RNA-seq measurements. Rather than inferring causal relationships between APA and miRNA stability, MIRNAPEX is designed as an analytical framework to interpret miRNA differential expression in the context of transcriptomic changes. In particular, it allows researchers to assess whether observed miRNA shifts are statistically consistent with changes in target abundance and 3′UTR isoform usage, thereby helping distinguish transcriptional from target landscape–associated contributions to miRNA variation. In addition, the framework can serve as a hypothesis-generating tool for perturbation studies affecting 3′UTR architecture, as illustrated here for TDMD trigger–miRNA pairs.

MIRNAPEX has some important limitations. It relies on bulk RNA-seq–derived APA metrics and selection of features based on predicted target sets, which are imperfect proxies for the true binding landscape. Bulk data also mix cell types and states, so shifts in composition could confound expression and 3′UTR usage. Furthermore, coefficients from regularized models help interpretation but do not explain causal effect sizes and cannot determine the directionality between biogenesis, decay, and target-site availability. Finally, curated TDMD interactions are incomplete and context-dependent, limiting ground-truth validation.

Integrating direct AGO-binding data with isoform-resolved APA profiles and more detailed predictors of site efficacy will sharpen our understanding of how target landscapes influence miRNA levels. Applying such analyses at single-cell or time course resolution could help separate cell-state effects from true regulatory changes, while controlled perturbations with matched mRNA, APA, and small-RNA measurements would provide stricter benchmarks for testing mechanistic hypotheses.

Our findings suggest that a more comprehensive view of miRNA regulation can be obtained when dynamic changes in target-site availability and decay processes are explicitly taken into account. Incorporating these dimensions has the potential to support interpretation of miRNA differential expression by incorporating target-site context, strengthen functional interpretations, and increase the reliability of biomarker discovery.

## MATERIALS AND METHODS

### Data collection

To train the ML models for predicting miRNA logFCs based on RNA sequencing data, we assembled data sets from The Cancer Genome Atlas (TCGA) (Cancer Genome Atlas Research Network et al. 2013). We downloaded all available mRNA and miRNA quantification data from TCGA and cross-referenced these samples with the TC3A database (Feng et al. 2018), a resource that applies the DaPars algorithm to TCGA RNA-seq data to quantify APA patterns (Xia et al. 2014). APA is represented by percentage of distal usage index (PDUI) values, which serve as a measure for distinguishing long and short 3′UTRs. PDUI values range between 0 and 1. For miRNA, we obtained isoform-level quantification files and mapped them to mature miRNA entries using miRBase annotations (Kozomara et al. 2019).

The final data set comprised 8460 samples with matched mRNA, miRNA, and APA profiles. Specifically, it includes TPM values for gene expression, mean RPM values for 2000 mature miRNAs, and PDUI values for between 1058 and 11,266 genes per cancer type. Because APA usage is influenced by gene expression and biological context, the number of genes with valid PDUI values varies across cancer types. In total, the data set spans 32 distinct TCGA cancer types and forms the basis for training the miRNA-specific ML models.

### Feature engineering and sample definition

For each miRNA, putative target genes were obtained from the microT database (Tastsoglou et al. 2023) and ranked according to their gene-level microT interaction scores. To systematically evaluate the impact of feature set size, we constructed multiple input variants per miRNA by selecting the top 25, 50, 75, 100, 250, 500, 750, 1000, and 2000 highest-scoring target genes that are reported to have APA measurements. To generate training examples for each miRNA, we first randomly split all available samples into training (80%) and test (20%) sets. For model evaluation, the training set was further divided into five folds for cross-validation (CV). Within each fold, samples were stratified into high and low expression groups based on the expression level of the respective miRNA. Each sample from a specific fold was then randomly paired with a sample from the opposite expression group within the same fold. This strategy enabled the creation of diverse sample pairs representing varying expression differences while preventing data leakage between training and validation subsets. For each generated sample pair, we computed gene-level differential features for all target genes. Specifically, we calculated the logFC in mRNA expression between the two samples and the corresponding difference in PDUI values (ΔPDUI) for APA usage. To avoid undefined values due to zero expression, a correction of +1 was applied prior to logFC calculation. ΔPDUI values range between −1 and 1, reflecting relative changes in distal polyadenylation site usage. Features are computed in the same way for the test and full training sets (Fig. 1).

### Training ML models to predict miRNA expression changes

We trained a range of ML algorithms to predict logFC values of mature miRNAs using the transcriptomic feature sets described above. To evaluate model performance under varying input conditions, we generated multiple training data sets based on different random pairings of samples and varying numbers of miRNA target genes (ranging from 25 to 2000). For each configuration, we trained both linear and nonlinear regression models implemented in the scikit-learn library (Pedregosa et al. 2011), including ordinary least squares (OLS), lasso (LA), ridge (RI), elastic net (EN), histogram-based gradient boosting (HB) regressor, random forest (RF) regressor, and multilayer perceptron (MLP). Hyperparameters for each model were optimized using CV within the training folds. For each miRNA, the model and hyperparameter combination that achieved the highest mean *R*^2^ across CVs was selected and retrained on the full training set to produce the final predictive model.

### The MIRNAPEX workflow

The resulting miRNA-specific ML models form the core of MIRNAPEX, enabling prediction of miRNA logFC values between two groups of RNA-seq samples. The MIRNAPEX pipeline automates the full process, starting from the raw FASTQ files. It integrates the GDC mRNA quantification pipeline (https://docs.gdc.cancer.gov/Data/Bioinformatics_Pipelines/Expression_mRNA_Pipeline/) and DaPars-based APA analysis (Xia et al. 2014; Feng et al. 2018; Li et al. 2021a) to compute gene-level logFC and ΔPDUI values for predefined miRNA target genes. These features are then passed to pretrained miRNA-specific regression models to predict logFC values for 1165 miRNAs across any two user-defined sample groups (Supplemental Fig. 1).

### APA perturbation

To test whether APA-driven changes in binding-site availability translate into shifts in mature miRNA levels, four RNA-seq comparisons of APA-regulator knockdowns with matched controls were analyzed. These perturbations remodel 3′UTRs, altering binding-site dosage. MIRNAPEX was then applied to predict miRNA log fold changes between perturbed and control samples, and concordance with APA-driven target-site changes was evaluated. The data sets involve CFIm25 knockdown in HCT116 (GSE158591) as CFIm25-KD-1 (Scarborough et al. 2021), CPSF6 knockdown in HEP3B (GSE229281) as CPSF6-KD (Sim et al. 2024), and the HEK293 experiments comprising an independent CFIm25 knockdown replicate and a CFIm68 knockdown (GSE179630) as CFIm25-KD-2 and CFIm68-KD (Ghosh et al. 2022), respectively. We validated mature miRNA expression levels in respective cell lines using DIANA-miTED (Kavakiotis et al. 2022) and annotated miRNA binding sites on target transcripts with predictions from the DIANA-microT (Tastsoglou et al. 2023).

To approximate transcriptional contributions to mature miRNA abundance, we quantified host-gene intronic RNA-seq signal for miRNAs with annotated host genes. Intronic intervals were inferred from annotated transcript exon structures and aggregated at the gene level to obtain a nonredundant set of host-gene intronic regions. Counts across these regions were summarized per condition and transcriptional proxy changes were reported as logFC (knockdown/control). Genome browser views of RNA-seq coverage across host-gene loci are provided in Supplemental Figures 2–6 to illustrate the intronic signal used as transcriptional proxy.

This study did not involve any new experiments on human participants or animals. All data were obtained from public databases, and therefore, no ethics approval was necessary.

## DATA DEPOSITION

The MIRNAPEX workflow is openly available at https://github.com/mcihan0bioinf/MIRNAPEX and archived on Zenodo (https://doi.org/10.5281/zenodo.17474139). It is provided as a fully defined computational pipeline within a conda environment. Model coefficients and training codes are accessible through this repository.

## SUPPLEMENTAL MATERIAL

Supplemental material is available for this article.

## Figures and Tables

**Mert Cihan:**
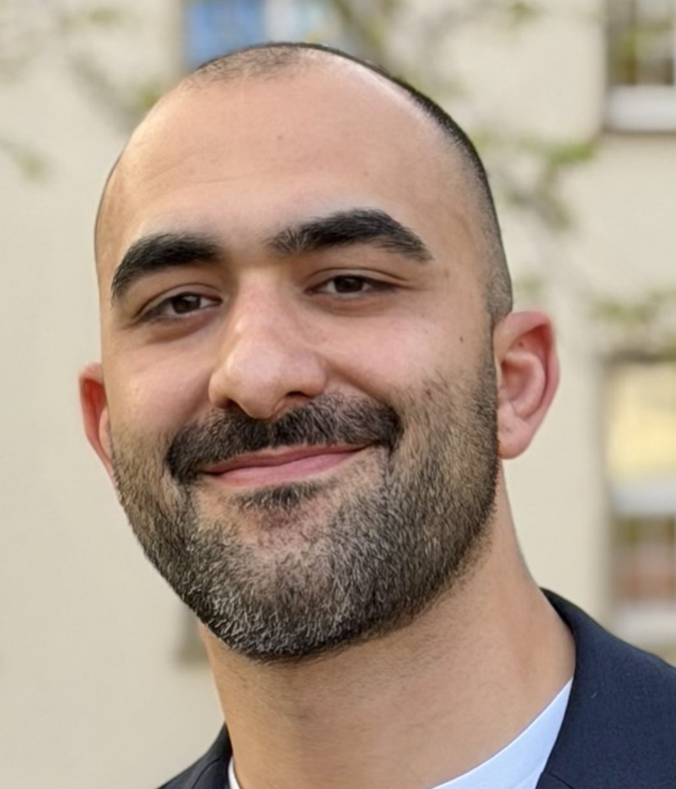


## MEET THE FIRST AUTHOR

***Meet the First Author(s)* is an editorial feature within *RNA*, in which the first author(s) of research-based papers in each issue have the opportunity to introduce themselves and their work to readers of *RNA* and the RNA research community. Mert Cihan is the first author of this paper, “Target-site dynamics explain a large share of apparent microRNA differential expression.” Mert is a PhD candidate in the Computational Biology and Data Mining Group led by Professor Miguel A. Andrade-Navarro at the Johannes Gutenberg University Mainz, Germany. His research focuses on studying systems-level properties intersecting with microRNA function through computational approaches.**

**What are the major results described in your paper, and how do they impact this branch of the field?**

We developed MIRNAPEX, a machine learning tool that predicts changes in microRNA levels directly from standard RNA-seq data. By combining gene expression with information about 3′UTR length, which determines how many microRNA binding sites a transcript carries, we found that a substantial portion of apparent microRNA differential expression can be traced back to changes in target-site availability. This was further supported by findings that shortening of 3′UTRs can reduce sites known to trigger microRNA decay. Our results suggest that microRNA expression changes may also reflect shifts in the transcriptomic landscape, and we hope this perspective encourages the community to consider target-site dynamics when interpreting microRNA differential expression results.

**What led you to study RNA or this aspect of RNA science?**

The availability of small RNA sequencing data remains limited compared to mRNA sequencing. This asymmetry motivated me to ask whether microRNA regulatory activity could be inferred directly from standard transcriptomic data. That question naturally led to alternative polyadenylation, because 3′UTR length changes dynamically modulate the binding sites available to microRNAs. Connecting these two layers, gene expression and 3′UTR architecture, into a unified predictive framework became the foundation of MIRNAPEX.

**What are some of the landmark moments that provoked your interest in science or your development as a scientist?**

Taking my first bioinformatics course during my undergraduate studies and realizing that biological questions could be addressed with code was a turning point. At the same time, I came to appreciate that the field of bioinformatics and computational biology allows one to engage with virtually any biological research question, from cancer genomics to RNA regulation to evolution. That realization, that I would never be restricted to a single system or organism but could follow my curiosity wherever the data led, is what drew me in and has kept me motivated ever since.

**If you were able to give one piece of advice to your younger self, what would that be?**

Don't be afraid to reach out to people. Science flourishes with communication.

**What are your subsequent near- or long-term career plans?**

I plan to pursue a postdoctoral position that integrates AI with single-cell and spatial transcriptomics in the context of small RNA regulation. The methods I developed during my PhD convinced me that microRNA activity can be read from transcriptomic landscapes, and I want to extend that idea to spatially resolved data, where the relationship between local 3′UTR architecture and small RNA function remains largely unexplored, particularly its influence on cancer stem cell fates.
